# Evidence of the Generation of Isosaccharinic Acids and Their Subsequent Degradation by Local Microbial Consortia within Hyper-Alkaline Contaminated Soils, with Relevance to Intermediate Level Radioactive Waste Disposal

**DOI:** 10.1371/journal.pone.0119164

**Published:** 2015-03-06

**Authors:** Simon P. Rout, Christopher J. Charles, Eva J. Garratt, Andrew P. Laws, John Gunn, Paul N. Humphreys

**Affiliations:** 1 Department of Biological Sciences, School of Applied Sciences, University of Huddersfield, Queensgate, Huddersfield, HD1 3DH, United Kingdom; 2 School of Geography, Earth and Environmental Sciences, University of Birmingham, Edgbaston, Birmingham, B15 2TT, United Kingdom; Chengdu Institute of Biology, CHINA

## Abstract

The contamination of surface environments with hydroxide rich wastes leads to the formation of high pH (>11.0) soil profiles. One such site is a legacy lime works at Harpur Hill, Derbyshire where soil profile indicated *in-situ* pH values up to pH 12. Soil and porewater profiles around the site indicated clear evidence of the presence of the α and β stereoisomers of isosaccharinic acid (ISA) resulting from the anoxic, alkaline degradation of cellulosic material. ISAs are of particular interest with regards to the disposal of cellulosic materials contained within the intermediate level waste (ILW) inventory of the United Kingdom, where they may influence radionuclide mobility via complexation events occurring within a geological disposal facility (GDF) concept. The mixing of uncontaminated soils with the alkaline leachate of the site resulted in ISA generation, where the rate of generation *in-situ* is likely to be dependent upon the prevailing temperature of the soil. Microbial consortia present in the uncontaminated soil were capable of surviving conditions imposed by the alkaline leachate and demonstrated the ability to utilise ISAs as a carbon source. Leachate-contaminated soil was sub-cultured in a cellulose degradation product driven microcosm operating at pH 11, the consortia present were capable of the degradation of ISAs and the generation of methane from the resultant H_2_/CO_2_ produced from fermentation processes. Following microbial community analysis, fermentation processes appear to be predominated by Clostridia from the genus *Alkaliphilus* sp, with methanogenesis being attributed to *Methanobacterium* and *Methanomassiliicoccus* sp. The study is the first to identify the generation of ISA within an anthropogenic environment and advocates the notion that microbial activity within an ILW-GDF is likely to influence the impact of ISAs upon radionuclide migration.

## Introduction

**Fig 1:**
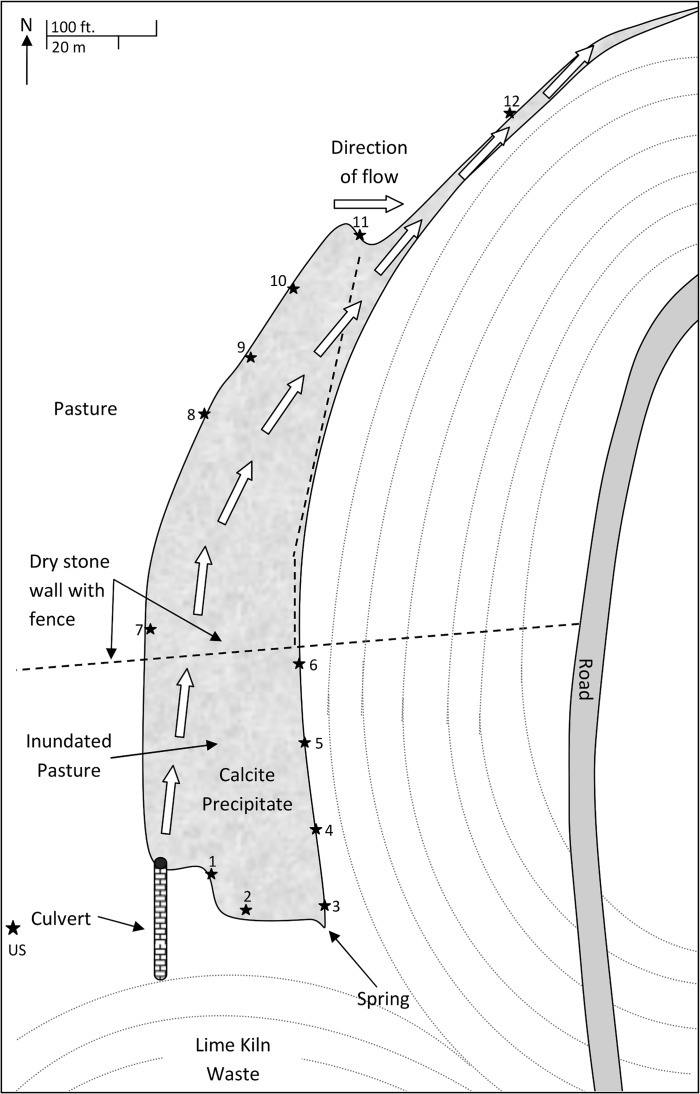
Site overview. One design concept for the long term storage of the United Kingdom’s intermediate level radioactive waste (ILW) inventory is a geological disposal facility (GDF) with a cementitious backfill [4]. Under the alkaline (10 < pH > 13), anoxic and saturated conditions predicted to develop following the closure of such a facility the cellulosic materials present in the waste [5] will undergo alkaline hydrolysis [6]. This process generates a range of organic compounds dominated by the α and β stereoisomers of isosaccharinic acid (ISA) (Fig. 2) [6,7]. ISA is of relevance to radioactive waste disposal since it is able to form complexes with certain radionuclides, enhancing their migration and consequently ISA may influence the performance of an ILW GDF [8,9].

Natural and anthropogenic hyper-alkaline environments have received considerable attention due to their potential as analogues for radioactive waste disposal sites [1–3]. One such example is the lime kiln waste site at Brookbottom, Harpur Hill, Derbyshire, UK (Fig. 1). Until 1944, a lime kiln was in operation adjacent to the site, generating a range of CaO containing wastes which were deposited at the southern end of the adjacent valley. Rainwater percolates through these wastes and generates an alkaline leachate (pH 12.0–13.0) which emerges from both an adit in the SW corner and a spring in the SE corner of the site. The leachate then flows in a N/NW direction following the path of the original brook. On emergence, the alkaline water absorbs atmospheric CO_2_, generating a tufa deposit which has infilled the valley floor and is encroaching on the adjacent farmland.

**Fig 2:**
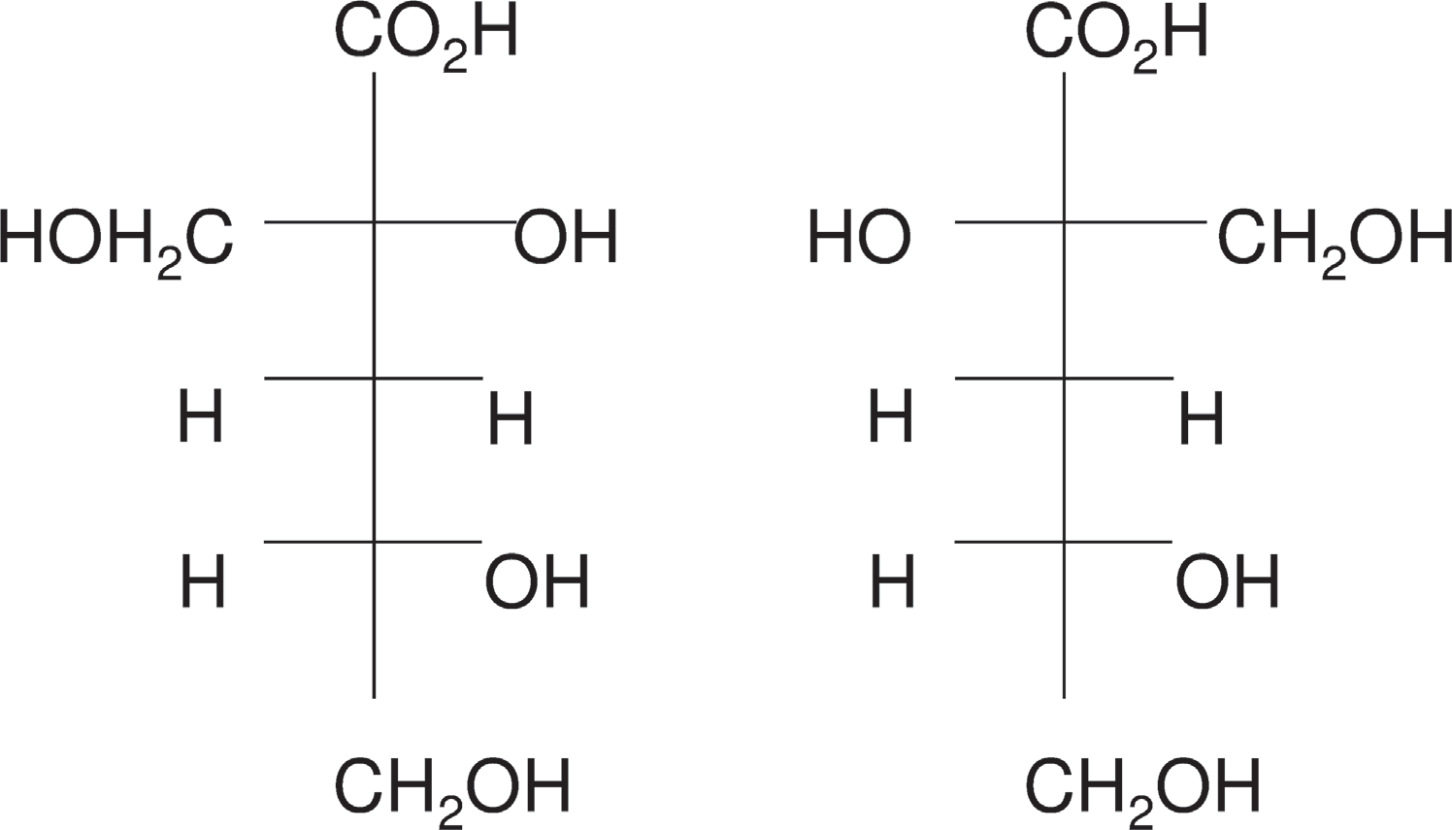
The α- (L) and β- (R) conformations of isosaccharinic acid (ISA). Although the presence of ISA is not observed in the natural environment, previous research has shown that micro-organisms from neutral sediments are capable of the utilisation of both α and β ISA within microcosm experiments. No significant difference in ISA degradation rates were observed across microcosms operating under iron reducing, sulphate reducing and methanogenic conditions at pH 7.5 [10]. When methanogenic sediments were subjected to further increases in pH (up to pH 10), the overall ISA degradation rate was slower. In addition, a difference was seen in the degradation rates of α and β ISA suggesting the potential for the persistence of the β stereoisomer within and surrounding the chemically disturbed zone of an ILW-GDF [11]. These communities were incapable of surviving at a pH of 11.0, suggesting that longer timescales may be required for micro-organisms to adapt to environmental pH values above pH 10.0, rather than adapting an ability to degrade ISA. In view of this, the hyper alkaline environment present at Harpur Hill provides an alkaliphilic soil dwelling microbial consortia which may be more indicative of the consortia that may develop with the near-field of an ILW-GDF.

Previous investigations of the microbial consortia at Harpur Hill have demonstrated the presence of anaerobic, alkaliphilic microbial populations within an organic rich soil layer at the site [12]. More recent work has demonstrated that these populations are capable of utilising α-ISA within 15 days under aerobic and nitrate reducing conditions, with modest utilisation under iron reducing conditions at pH 10 [13]. As oxygen is likely to be depleted post closure due to corrosion processes and the presence of nitrate and ferric iron will be limited, fermentative and methanogenic processes are likely to play a more important role in the fate of ISA within an ILW-GDF. The aim of this study was to determine whether or not the stereoisomers of ISA are generated within the Harpur Hill site at the interface between hyper alkaline leachate and the soil zone due to the alkaline hydrolysis of cellulosic materials present in the soil. If ISA is generated *in-situ*, the resident microbial consortia represents a source of alkali-tolerant and alkaliphilic micro-organisms that may provide an insight into the impact of microbial activity on the transport and complexation of radionuclides within an ILW-GDF.

## Material and Methods

### Field sampling

In April 2013, twelve hand cored samples (ca. 5 cm diameter, and 5 cm depth) were taken around the periphery of the site (Fig. 1, 53° 14’ 8.4732” N, 1° 55’ 1.2648” W), which was accessed from the public right of way adjacent to the site. As an orphaned site, no specific permissions were required to sample and the field studies did not involve endangered or protected species. Samples 1 to 6 were taken from regions where an established tufa deposit was present and in these cases the tufa was removed prior to sampling. Samples 7 to 12 were taken from regions where grassland had been recently inundated, as indicated by adjacent vegetation. In addition, an uncontaminated sample was taken away from the waste filled region (Fig. 1, US). A 20L sample of the alkaline leachate (pH 13.6) flowing from the brick culvert was collected and stored at 4°C in the absence of air. Soil pH was measured on site using a portable pH meter and calibrated electrodes (Mettler Toledo, UK). In addition, the pH of the collected sample was assessed post removal using British standard ISO 10390:2005 [14]. The Eh of the samples was measured using an InLab Redox Micro probe (Mettler Toledo, UK).

Total ISA was extracted from the soil samples via the addition of 5 mL of 2 M hydrochloric acid to 1 g soil sample, such that the resultant pH was <pH 4.0. The mixture was vortexed and left overnight before centrifugation (10 min, 8000 x*g*) to remove any solids. Water was then removed using rotary evaporation at 80°C and the resultant solids were dissolved in ultra-pure water to a concentration of 1000 mg/L prior to analysis. ISA analysis was performed using High Performance Anion Exchange Chromatography (Dionex, Camberly, UK) employing Pulsed Amperometric Detection (HPAEC-PAD) and a Dionex Carbopac PA20 column (3 x 150 mm, 6.5 μm particle size) eluted with aqueous sodium hydroxide (0.05 mol L^−1^) alongside a range of previously synthesised standards [15]. A second soil sample was also centrifuged at 8000 x*g* for 30 min to separate the sediment from the pore water. The pore water was then syringe filtered through a sterile 0.45 μm filter (Sartorius, UK) and stored at 4°C prior to use. The presence of soluble isosaccharinic acid was measured using HPAEC-PAD as outlined above. Volatile fatty acids were extracted from the soil samples using a standard method [16] and analysed via gas chromatography with flame ionisation detection (GC-FID) equipped with a HP-FFAP column (30 m x 0.535 m x 1.00 μm; Agilent Technologies). Samples (1 μL) were passed through the column under the following conditions: 95°C (2 min) to 140°C at 10°C min^−1^, then to 200°C (held 10 min) at 40°C min^−1^. Total Fe (III) and Fe (II) were measured using the ferrozine extraction method described previously [17]. Nitrate and sulphate content were measured via ion chromatography using amperometric detection and a Metrohm 850 Professional IC (Metrohm, Cheshire, UK) employing a Metrohm Metrosep A Supp 5 column (4 x 150mm, 5 μm particle size) and eluting with sodium carbonate and sodium hydrogen carbonate (3.2 mmol L^−1^, 1.0 mmol L^−1^ respectively) alongside a range of standards.

### ISA generation from uncontaminated soil

A range of reaction vessels were established in 100 mL rubber butyl stoppered glass bottles, in which uncontaminated soil was mixed 5% w/v with filtered (0.22 μm filter unit, Millipore, US) alkaline leachate (pH 13.6) or leachate adjusted to pH 7.5 using conc HCl. In addition, both the alkaline and pH adjusted leachates were mixed with uncontaminated soil that had been double autoclaved prior to use. All reaction vessels were prepared within an anaerobic chamber (Bugbox, Don Whitely, UK). Reaction vessels were prepared in triplicate (9 per reaction condition) and incubated at 4, 10 or 20°C. Samples (1 mL) were taken every 4 weeks, centrifuged at 8000 x*g* for 2 minutes and filtered using a 0.45μm filter prior to ISA analysis using HPAEC-PAD.

### Microcosm experiments

A soil sample (50 g) from Site 6 (Fig. 1) was suspended in 250 mL of anaerobic mineral media along with 50 mL of synthesised alkaline cellulose degradation products (CDP) [10] and incubated at 25°C in a stirred reaction vessel as previously described [18]. Immediately after its preparation the pH of the microcosm was adjusted to pH 11.0 using 4 M NaOH. The solution was then purged with nitrogen for 30 minutes prior to the vessel being sealed. The microcosm was then fed with 50 mL of the CDP under a stream of nitrogen every 14 days, until the total culture volume reached 500 mL (56 days). Once the working volume of 500 mL was reached, the microcosm was switched to a feed and waste cycle where 50 mL of microcosm contents was replaced by 50 mL of CDP every 14 days. Microcosm pH was amended to pH 11.0 with 4 M NaOH at the beginning of each feed cycle for the duration of the investigation. This regime was repeated for a further 70 days to allow for the stabilisation of the chemistry within the reactor. After stabilisation samples were taken over the course of a further 3 consecutive feeds (42 days). Samples were centrifuged at 8,000 x *g* for 1 minute to remove solids and filtered through a sterile 0.45 μm filter to remove any microorganisms before being stored at −20°C prior to analysis. Filtrate was used for ISA analysis using HPAEC-PAD and 900 μL of the filtrate was added to 100 μL of phosphoric acid prior to volatile fatty acid analysis using GC-FID as previously described. Methane present in the microcosm headspace was detected by a gas sensor (BCP-CH_4_), connected to BACCom12 multiplexer utilising BACVis software (BlueSens gas sensor GmbH, Herten, Germany).

In addition, a set of control reactors were prepared in three rubber butyl stoppered 100 mL Wheaton bottles and amended with chloramphenicol to a concentration of 50 μg mL^−1^. The ISA and volatile fatty acid concentrations within these control reactors were monitored using the above methods. Headspace gas analysis was carried out using gas chromatography with thermal conductivity detection (GC-TCD) utilising a HP GS-Q column (30 m x 0.53 m x 1.00 μm; Agilent Technologies) with an oven temperature of 35°C.

### Amplification cloning, and sequencing of 16S rRNA gene sequences

Total genomic DNA was extracted from the soil microcosm using a Powersoil DNA extraction kit (Mo-BIO, Carlsbad, US). A ∼1500 bp fragment of the eubacterial 16S rRNA gene was amplified using broad specificity primers pA and pH as previously described [19] and a ∼750 bp fragment of the archaeal 16S rRNA gene was amplified using primers Ar and Af [20]. PCR reaction mixture contained 5 μL of extracted DNA, 1.5 μL of each primer (10pmol μL^−1^ concentration), and 25 μL of BIOMIX red master mix (BIOLINE, UK) made up to 50 μL volume with PCR grade water. The reaction mixture was then incubated at 94°C for 5 mins, and then cycled 35 times through three steps: denaturing (94°C, 1 min), annealing (60°C, 1min), primer extension (72°C, 1min 30s). This was followed by a final extension step of 72°C for 5 minutes. PCR was verified by electrophoresis of 5 μL samples of product in a 1.0% agarose TAE gel with ethidium bromide staining. The remaining 45 μL of product was purified using a Qiaquick PCR purification kit (Qiagen, UK). PCR products were ligated into the standard cloning vector PGEM-T easy (Promega, US) and transformed into E.coli JM109 competent cells (Promega, US). Transformed cells were grown on Luria Bertani (LB) agar containing 100μg mL^−1^ ampicillin overlaid with 40 μL of 100 mM IPTG and 40 μL of 40mg mL^−1^ X-GAL (5-bromo-4-chloro-3-indolyl-β-D-galactopyranoside) in N’N dimethylformamide for blue-white colour screening for 16 hours at 37°C. Insert containing colonies were sub-cultured on to LB plates containing ampicillin/IPTG/X-GAL as described previously and incubated for 24 hours at 37°C. Colonies were then transferred to 96 well plates containing LB agar with 150 mg mL^−1^ ampicillin and sequenced using Sanger sequencing technology (GATC Biotech, Germany). Inserts were amplified using a universal M13 forward primer as a sequencing start point. The resulting 16S rRNA gene sequences were grouped into Eubacterial and Archaeal divisions. The sequences were then aligned using the MUSCLE-Multiple Sequence Alignment package (http://www.ebi.ac.uk/Tools/services/web/toolform.ebi?tool=muscle). Aligned sequences were then chimera checked using Mothur [21] and sequences analysed against the NCBI database using Basic Local Alignment Search Tool (MegaBLAST) utilising the 16S ribosomal RNA sequences for Bacteria and Archaea. Sequences were then placed into phylogenetic families based on the closest sequence from the MegaBLAST output.

### Nucleotide sequence accession numbers

The 16S rRNA sequence data have been submitted to GenBank under accession numbers KP054397-KP054455.

### Statistical analysis

All statistical analysis was carried out using IBM SPSS V 20 for Windows. Bivariate correlations were determined by calculating Pearson correlation coefficient.

## Results and Discussion

### Site survey

ISA was detected in the porewater and sediments from sample sites where an alkaline pH predominated (Table 1). ISA extracted from the sediments was solely in the α conformation, reflecting the limited solubility of the Ca^2+^ salt of α-ISA at high pH [22,23]. The greater solubility of β-ISA under high pH, calcium rich conditions is reflected in its absence from sediment samples; although some β-ISA was detectable in soil porewaters. The α-ISA was most abundant in samples which had been subjected to longer term tufa contamination (S1–6; Fig. 1) rather than those most recently inundated (S7–12). The uncontaminated soil sample showed no evidence of ISA generation following acid extraction. The surrounding porewaters also contained volatile fatty acids throughout sites 1–12 in varying concentrations alongside a range of terminal electron acceptors able to support a wide range of microbial processes, with nitrate, sulphate and reducible iron present across the entire site as observed by others [12,24].

**Table 1:** Pore water and soil analysis from twelve sample sites around the Brookfoot site, Harpur Hill, Buxton, UK.

Site	Porewater Analyses										Soil analysis		
	pH	μmol L^−1^									pH	Eh	μmol g^−1^
		Fe(II)	Fe(III)	α-ISA	β-ISA	X-ISA	Acetic Acid	Other VFA	Sulphate	Nitrate			α-ISA
1	12.14	2.99	2.29	16.70	27.70	151.71	190.36	58.07	279.27	31.13	11.70	−62.00	15.78
2	7.80	0.03	14.19	0.00	6.88	37.67	248.24	248.52	8.96	8.55	7.70	−66.00	28.78
3	12.50	0.80	3.98	5.06	3.28	18.21	0.00	148.70	626.88	1267.10	11.20	−49.00	344.72
4	12.40	0.00	0.98	0.00	0.00	45.70	0.00	95.34	121.88	258.87	11.07	−43.00	111.72
5	12.70	0.00	1.43	5.26	8.35	0.00	0.00	240.65	555.94	1809.19	11.35	−47.00	0.07
6	12.10	1.10	2.34	16.43	39.67	96.96	0.00	122.78	103.85	110.32	10.80	−34.00	0.13
7	12.40	4.84	7.59	0.00	21.90	0.00	1102.21	214.32	162.92	5.16	10.17	−74.00	0.79
8	12.10	0.00	5.17	0.00	0.00	0.00	0.00	134.08	186.98	2.90	10.30	−89.00	0.21
9	8.00	0.07	5.55	10.72	45.82	250.73	815.26	568.25	77.19	4.68	8.00	−42.00	0.22
10	11.20	1.83	9.03	0.00	0.00	0.00	0.00	152.29	58.96	4.68	9.00	−26.00	4.44
11	8.10	0.00	6.06	0.00	0.00	0.00	0.00	176.10	169.27	0.00	7.85	−25.00	0.21
12	11.50	0.00	5.90	0.00	0.00	0.00	0.00	178.17	36.98	98.39	10.30	43.00	0.22

### ISA generation from uncontaminated soil

**Fig 3:**
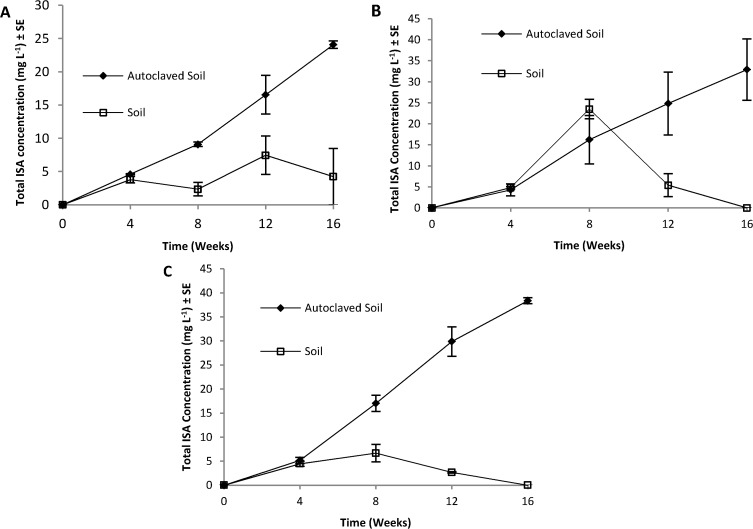
Presence of ISA’s abiotic reactions (closed diamond) and biotic reactions (open squares) involving uncontaminated soil and alkaline leachate from the site at 4°C (A), 10°C (B) and 20°C (C, n = 3).

**Fig 4:**
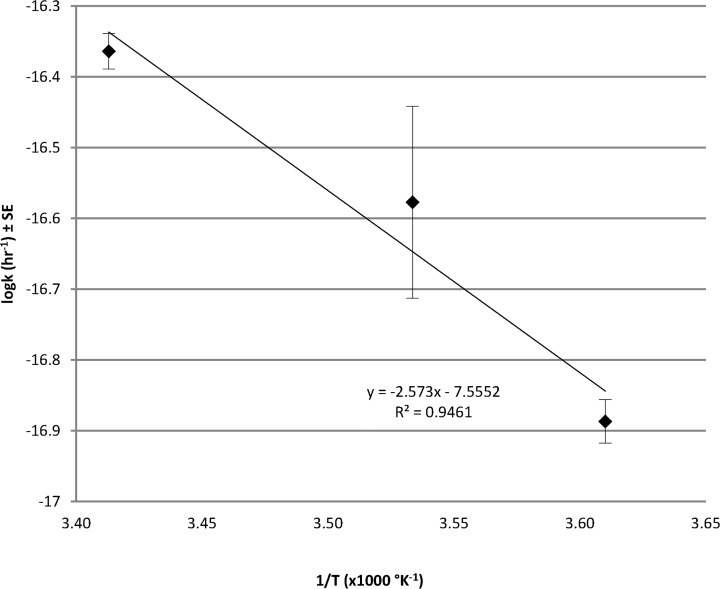
Arrhenius plot of aqueous ISA generation from uncontaminated soils in alkaline leachate.

The results of combining alkaline leachate with uncontaminated soil from the area surrounding the site can be seen in Fig. 3. In abiotic experiments, where soil was double autoclaved prior to the addition of the alkaline leachate, ISA was generated at all three temperatures across the 16 weeks of sampling. In contrast, in the presence of neutralised alkaline leachate no ISA was generated within the 16 weeks of sampling (data not shown). The rate of ISA generation increased with temperature, as observed by other authors employing pure cellulose and sodium hydroxide [25]. An Arrhenius plot of the calculated rates (Fig. 4), where statistical analysis showed a correlation between temperature and rate (p<0.05) and allowed the activation energy (21.4 J mol^−1^) of ISA generation to be calculated. This value is lower than reported values [25] for the propagation of the peeling reaction with cellulose and 1.25 M NaOH, which may reflect the degree of amorphism of the cellulosic materials present within the soils. In the biotic experiments where soil had not been autoclaved, generated ISA was either partially (4°C) or completely removed (10 and 20°C) by the end of 16 weeks of sampling. Indicating that temperature influenced both the rate of ISA generation and its subsequent microbial degradation.

### Microcosm Chemistry

**Fig 5:**
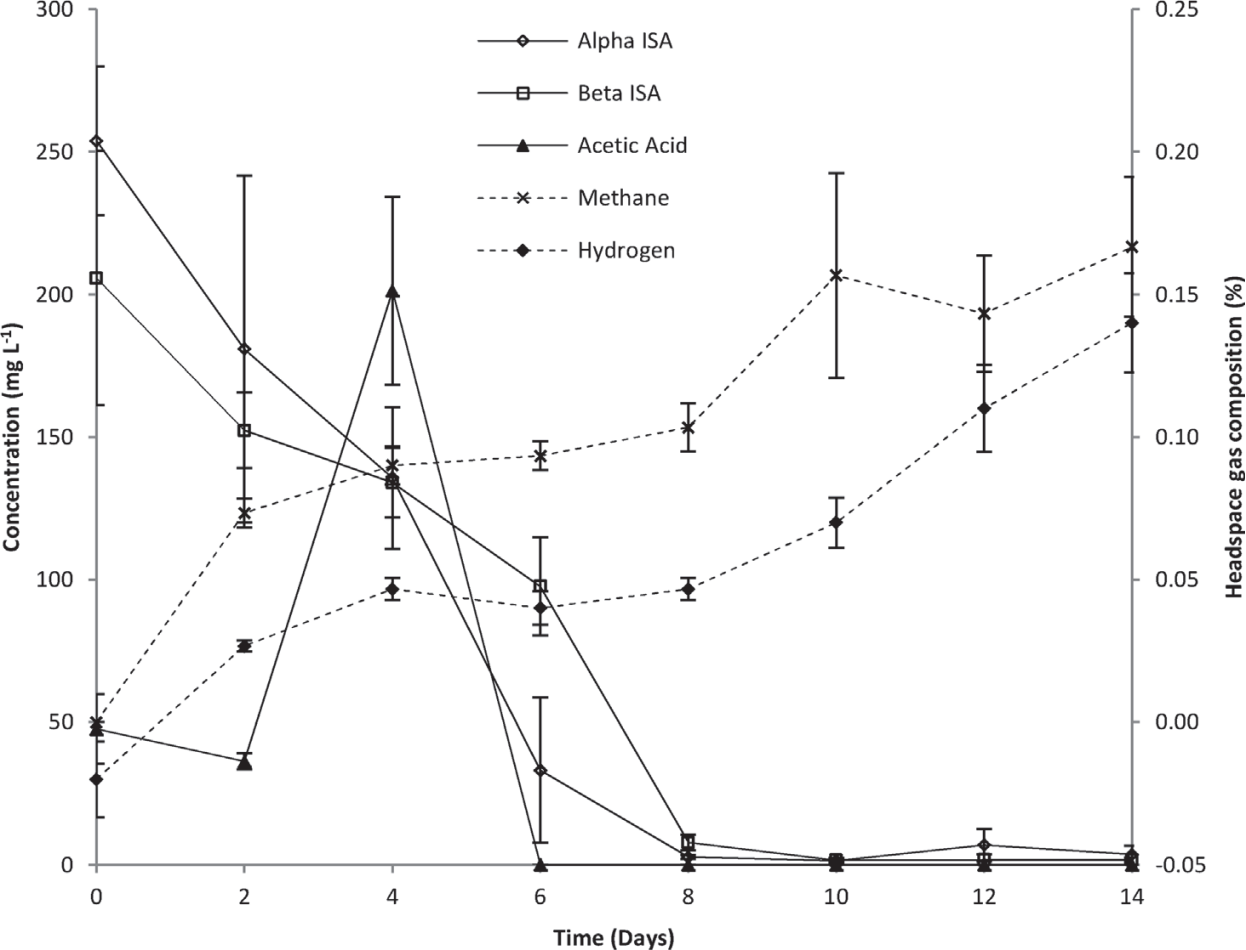
Chemistry of CDP driven microcosms operating at pH 11.0 using site contaminated soil as a starting inoculum.

The batch fed microcosm established using soil collected from the site (Fig. 5) demonstrated significant ISA degradation at pH 11.0. The microcosm demonstrated mean first order degradation rates for the individual stereoisomers of ISA of 1.69 x 10^−1^ day^−1^ (SE 3.28 x 10^−2^) for α-ISA and 1.13 x 10^−1^ day^−1^ (SE 1.07 x 10^−2^) for β-ISA. These rates are greater than those observed with consortia obtained from neutral sediments operating at pH 10 [11], indicating the greater degree of alkaline adaption seen at the Harpur Hill site. The microcosm consortia fermented ISA to hydrogen and acetic acid of which the latter was subsequently removed (Fig. 5). Methane accumulated within the headspace at a consistent rate after day 2 and its production did not correlate directly with the generation and removal of acetic acid (Fig. 5). Similarly, no clear trend could be observed with regards to the generation and removal of hydrogen within the system linked to the formation of methane. Carbon dioxide from fermentation processes is likely to precipitate as a carbonate within the alkaline conditions of the microcosm. Previous authors have noted the ability of methanogens to utilise calcite as a carbon source, suggesting that the rate of hydrogenotrophic methanogenesis is impacted by the availability of carbon dioxide [26], In samples amended with chloramphenicol, removal of ISAs from solution was not evident, the presence of VFAs was limited to those present within the CDP liquor and methane was not detected in the headspace gas. This indicates that the ISA removal observed is microbially mediated rather than being the result of sorption or chemical degradation processes (supporting information, Figs. A and B in S1 File).

### 16S rRNA gene clone libraries

**Fig 6:**
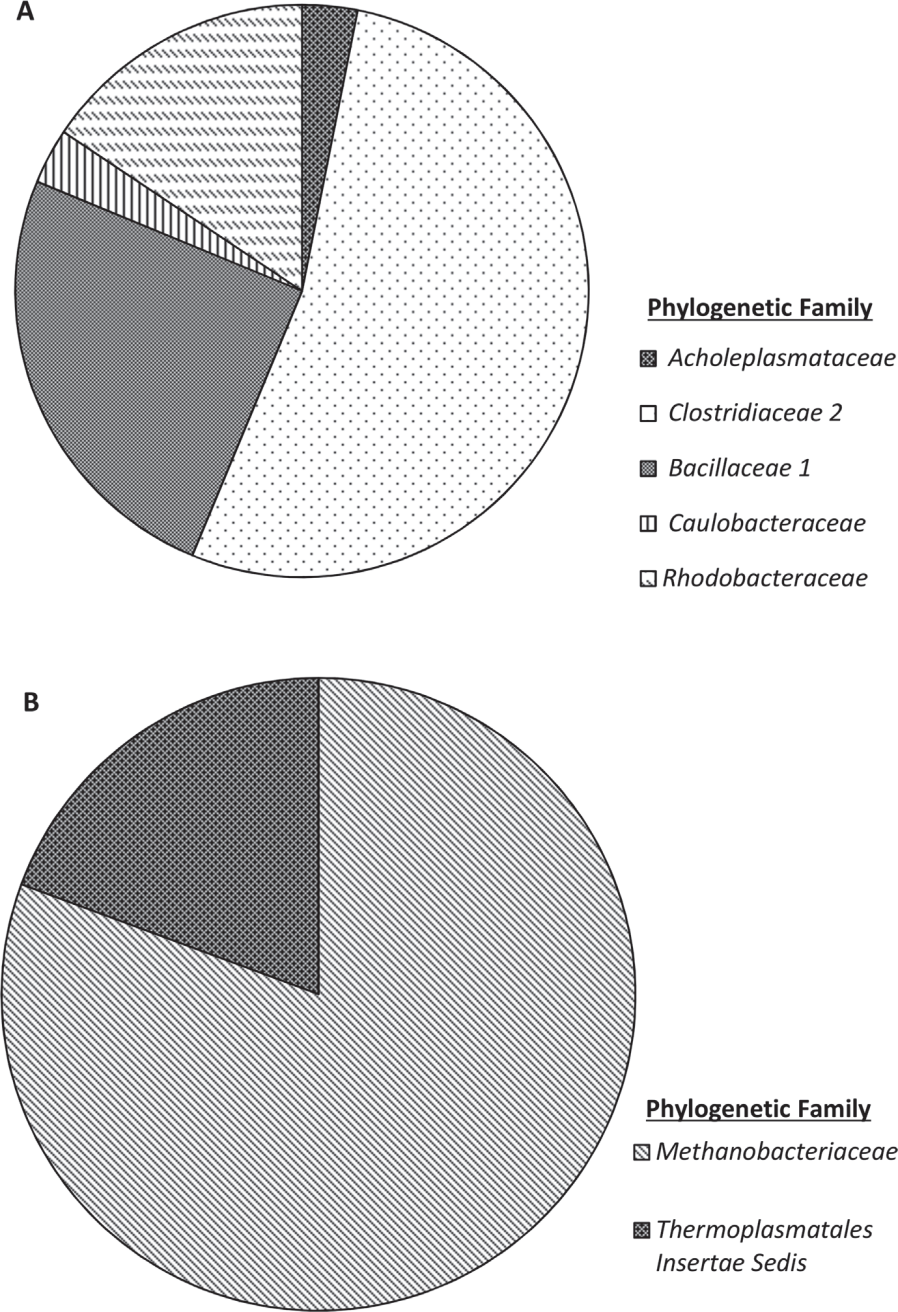
Eubacterial (A, n = 32) and Archaeal (B, n = 31) 16S rRNA gene clone libraries of pH 11 CDP driven microcosm where clones were assigned to a family based on the closest sequence match obtained through MegaBLAST database search.

The closest sequence matches for the Eubacterial clone library are presented within the supporting information (Table A in S1 File). The taxonomic composition of the 16S rRNA gene clone library is presented in Fig. 6A. 33 Eubacterial 16S rRNA gene sequences were obtained, of which 53% were most closely associated with the Family *Clostridiaceae* 2. Within this family, all the sequences were most closely related to sequences from the genera *Alkaliphilus*, with 8 sequences most closely matching *Alkaliphilus crotonatoxidans* strain B11-2, 7 sequences most closely matching *Alkaliphilus metalliredigens* strain QYMF and 2 most closely matching *Alkaliphilus transvaalensis* strain SAGM1. The isolation of this genera from hyperalkaline sites is well documented, as is their ability to metabolise a range of carbon sources [27–31], and as a wider class, *Clostridia* are well documented in their ability for carry out fermentation processes [10,32]. Of the remaining clones, 25% most closely matched organisms from the family *Bacillaceae* 1, where sequences most closely matched *Anaerobacillus alkalilacustris* strain Z-0521 (3 sequences), *Bacillus alcalophilus* strain NBRC 15653 (2 sequences), and *Bacillus okhensis* strain Kh10-101 (3 sequences). Much like the *Clostridiaceae* 2, the organisms observed here are also capable of metabolising carbohydrates and sugars as a carbon source under anaerobic conditions [33,34]. The remaining sequences most closely matched organisms belonging to the class *α-proteobacteria*, which are associated with a range of processes [35,36].

Closest sequence matches for the Archaeal clone library can be found in the supporting information (Table B, S1 File). The taxonomic composition is represented in Fig. 6B. In this case 81% of the clone library (n = 31), was represented by organisms from the family *Methanobacteriaceae* where 12 sequences most closely matched *Methanobacterium alcaliphilum* strain NBRC 105226 with the remaining sequences from this family (13 sequences) most closely matching *Methanobacterium flexile* strain GH. Both of these strains are hydrogenotrophic Archaea and have been shown to be phylogenetically related to one another [37], *M*. *alcaliphilum* has been isolated from alkaline sediments in Egypt with optimal growth conditions of pH 9.9 under laboratory conditions [38]. At the time of writing, *M*. *flexile* has only been isolated from mesophilic, neutral pH lake sediments, where no further testing of pH tolerances was carried out [37]. The 6 remaining sequences from the clone library most closely matched hydrogenotrophic methanogen *Methanomassiliicoccus luminyensis* strain B10 from the family *Thermoplasmatales insertae sedis*. Again, this hydrogenotrophic methanogen has been more commonly associated with the digestive tract of other organisms [39], and information regarding its tolerance of alkaline conditions are not available in the wider literature. The obligate hydrogenotrophic nature of the Archaeal clone library contradicts the removal of acetic acid seen within the system. This acetic acid metabolism may be linked to assimilation/anabolism by *Methanobacterium* sp [40,41], or degradation by Eubacteria within the consortia, where *Alkaliphilus* sp and *α—proteobacteria* are capable of acetate utilisation [42,43]. The latter would require an as yet unidentified terminal electron acceptor to be present within the system.

## Conclusion

This survey of the hyper-alkaline site at Harpur Hill, represents the first demonstration of the *in-situ* generation of both the α and β forms of ISA in terrestrial environments through the hydrolysis of soil organic material by anthropogenic alkaline leachates. Sediments at the site contain active microbial consortia able to ferment both forms of ISA with the subsequent generation of acetic acid, hydrogen and methane at a pH of 11.0. Molecular analysis of these consortia indicates that they are dominated by alkaliphilic Clostridia and hydrogenotrophic *Methanobacteriaceae*. These observations suggest that ISA may act as a key electron donor supporting the diverse subsurface microbial population previously observed at this site [12,13,44].

Regarding ILW disposal, this survey suggests that microbial populations able to degrade ISA may evolve within decades of site closure provided that the ambient pH is in the region of pH 11.0. Current estimates for near-field pH of a cementitious GDF suggest that the ambient pH will remain above pH 12.0 for hundreds to thousands of years [4], suggesting that ISA degrading consortia will be confined to lower pH regions within the waste or in the alkaline disturbed zone surrounding the GDF. Provided microbial activity is not inhibited by the ambient pH these results suggest that a microbial population similar to that which has evolved at Harpur Hill will be able to metabolise both the α and β forms of ISA generated within the site and in so doing mitigate the impact that these complexing will have on the transport of radionuclides.

## Supporting Information

S1 File Fig. A: Fate of acetate in biotic (open triangles) and abiotic (open squares) experiments. Fig. B: Fate of ISA in biotic (open triangles) and abiotic (open squares) experiments. Table A: Eubacterial clone library sequence matches. Table B: Archaeal clone library sequence matches.
(DOCX)
