# Supplementary material for: Evidence of the Generation of Isosaccharinic Acids and Their Subsequent Degradation by Local Microbial Consortia within Hyper-Alkaline Contaminated Soils, with Relevance to Intermediate Level Radioactive Waste Disposal
Source: PLoS One. 2015 Mar 6;10(3):e0119164. doi: 10.1371/journal.pone.0119164 (PMC4351885; doi:10.1371/journal.pone.0119164)
Supplement: S1 File — Fig. B: Fate of ISA in biotic (open triangles) and abiotic (open squares) experiments. Table A: Eubacterial clone library sequence matches. Table B: Archaeal clone library sequence matches. (DOCX) [file pone.0119164.s001.docx]

Figure A: Fate of acetate in biotic (open triangles) and abiotic (open squares) experiments

Figure B: Fate of ISA in biotic (open triangles) and abiotic (open squares) experiments

| **Clone ID** | **Closest Sequence Match** | **Similarity** |
| --- | --- | --- |
| METHEUB1 | Bacillus alcalophilus strain NBRC 15653 16S ribosomal RNA gene, partial sequence | 94 |
| METHEUB2 | Bacillus alcalophilus strain NBRC 15653 16S ribosomal RNA gene, partial sequence | 94 |
| METHEUB3 | Bacillus okhensis strain Kh10-101 16S ribosomal RNA gene, partial sequence | 96 |
| METHEUB4 | Alkaliphilus transvaalensis strain SAGM1 16S ribosomal RNA gene, partial sequence | 90 |
| METHEUB5 | Bacillus okhensis strain Kh10-101 16S ribosomal RNA gene, partial sequence | 96 |
| METHEUB6 | Anaerobacillus alkalilacustris strain Z-0521 16S ribosomal RNA gene, partial sequence | 96 |
| METHEUB7 | Alkaliphilus crotonatoxidans strain B11-2 16S ribosomal RNA gene, partial sequence | 97 |
| METHEUB8 | Alkaliphilus crotonatoxidans strain B11-2 16S ribosomal RNA gene, partial sequence | 97 |
| METHEUB9 | Roseicitreum antarcticum strain ZS2-28 16S ribosomal RNA gene, partial sequence | 90 |
| METHEUB10 | Octadecabacter arcticus strain 238 16S ribosomal RNA gene, complete sequence | 83 |
| METHEUB11 | Roseicitreum antarcticum strain ZS2-28 16S ribosomal RNA gene, partial sequence | 91 |
| METHEUB12 | Alkaliphilus metalliredigens strain QYMF 16S ribosomal RNA gene, complete sequence | 97 |
| METHEUB13 | Alkaliphilus crotonatoxidans strain B11-2 16S ribosomal RNA gene, partial sequence | 97 |
| METHEUB14 | Roseicitreum antarcticum strain ZS2-28 16S ribosomal RNA gene, partial sequence | 90 |
| METHEUB15 | Alkaliphilus metalliredigens strain QYMF 16S ribosomal RNA gene, complete sequence | 94 |
| METHEUB16 | Alkaliphilus metalliredigens strain QYMF 16S ribosomal RNA gene, complete sequence | 97 |
| METHEUB17 | Alkaliphilus metalliredigens strain QYMF 16S ribosomal RNA gene, complete sequence | 94 |
| METHEUB18 | Alkaliphilus transvaalensis strain SAGM1 16S ribosomal RNA gene, partial sequence | 90 |
| METHEUB19 | Bacillus okhensis strain Kh10-101 16S ribosomal RNA gene, partial sequence | 95 |
| METHEUB20 | Alkaliphilus metalliredigens strain QYMF 16S ribosomal RNA gene, complete sequence | 94 |
| METHEUB21 | Alkaliphilus metalliredigens strain QYMF 16S ribosomal RNA gene, complete sequence | 97 |
| METHEUB22 | Alkaliphilus metalliredigens strain QYMF 16S ribosomal RNA gene, complete sequence | 94 |
| METHEUB23 | Alkaliphilus crotonatoxidans strain B11-2 16S ribosomal RNA gene, partial sequence | 97 |
| METHEUB24 | Roseicitreum antarcticum strain ZS2-28 16S ribosomal RNA gene, partial sequence | 90 |
| METHEUB25 | Anaerobacillus alkalilacustris strain Z-0521 16S ribosomal RNA gene, partial sequence | 96 |
| METHEUB26 | Alkaliphilus crotonatoxidans strain B11-2 16S ribosomal RNA gene, partial sequence | 97 |
| METHEUB27 | Acholeplasma parvum strain H23M 16S ribosomal RNA gene, partial sequence | 90 |
| METHEUB28 | Alkaliphilus crotonatoxidans strain B11-2 16S ribosomal RNA gene, partial sequence | 97 |
| METHEUB29 | Brevundimonas diminuta strain ATCC 11568 16S ribosomal RNA gene, complete sequence | 99 |
| METHEUB30 | Alkaliphilus crotonatoxidans strain B11-2 16S ribosomal RNA gene, partial sequence | 97 |
| METHEUB31 | Alkaliphilus crotonatoxidans strain B11-2 16S ribosomal RNA gene, partial sequence | 97 |
| METHEUB32 | Anaerobacillus alkalilacustris strain Z-0521 16S ribosomal RNA gene, partial sequence | 96 |

Table A: Eubacterial clone library sequence matches

| **Clone ID** | **Closest Sequence Match** | **Similarity** |
| --- | --- | --- |
| METHARC2 | Methanobacterium alcaliphilum strain NBRC 105226 16S ribosomal RNA gene, partial sequence | 99 |
| METHARC3 | Methanobacterium alcaliphilum strain NBRC 105226 16S ribosomal RNA gene, partial sequence | 99 |
| METHARC4 | Methanobacterium alcaliphilum strain NBRC 105226 16S ribosomal RNA gene, partial sequence | 99 |
| METHARC5 | Methanomassiliicoccus luminyensis strain B10 16S ribosomal RNA gene, partial sequence | 92 |
| METHARC7 | Methanobacterium flexile strain GH 16S ribosomal RNA gene, partial sequence | 99 |
| METHARC9 | Methanobacterium flexile strain GH 16S ribosomal RNA gene, partial sequence | 99 |
| METHARC10 | Methanobacterium flexile strain GH 16S ribosomal RNA gene, partial sequence | 99 |
| METHARC11 | Methanobacterium alcaliphilum strain NBRC 105226 16S ribosomal RNA gene, partial sequence | 99 |
| METHARC12 | Methanobacterium alcaliphilum strain NBRC 105226 16S ribosomal RNA gene, partial sequence | 99 |
| METHARC13 | Methanobacterium flexile strain GH 16S ribosomal RNA gene, partial sequence | 99 |
| METHARC14 | Methanobacterium flexile strain GH 16S ribosomal RNA gene, partial sequence | 99 |
| METHARC15 | Methanobacterium flexile strain GH 16S ribosomal RNA gene, partial sequence | 100 |
| METHARC16 | Methanobacterium flexile strain GH 16S ribosomal RNA gene, partial sequence | 99 |
| METHARC17 | Methanobacterium flexile strain GH 16S ribosomal RNA gene, partial sequence | 99 |
| METHARC20 | Methanobacterium flexile strain GH 16S ribosomal RNA gene, partial sequence | 99 |
| METHAR25 | Methanomassiliicoccus luminyensis strain B10 16S ribosomal RNA gene, partial sequence | 89 |
| METHARC28 | Methanomassiliicoccus luminyensis strain B10 16S ribosomal RNA gene, partial sequence | 89 |
| METHARC29 | Methanomassiliicoccus luminyensis strain B10 16S ribosomal RNA gene, partial sequence | 89 |
| METHARC31 | Methanobacterium alcaliphilum strain NBRC 105226 16S ribosomal RNA gene, partial sequence | 99 |
| METHARC34 | Methanobacterium alcaliphilum strain NBRC 105226 16S ribosomal RNA gene, partial sequence | 99 |
| METHARC35 | Methanomassiliicoccus luminyensis strain B10 16S ribosomal RNA gene, partial sequence | 89 |
| METHARC36 | Methanobacterium alcaliphilum strain NBRC 105226 16S ribosomal RNA gene, partial sequence | 99 |
| METHARC37 | Methanobacterium flexile strain GH 16S ribosomal RNA gene, partial sequence | 99 |
| METHARC38 | Methanobacterium alcaliphilum strain NBRC 105226 16S ribosomal RNA gene, partial sequence | 99 |
| METHARC40 | Methanobacterium alcaliphilum strain NBRC 105226 16S ribosomal RNA gene, partial sequence | 99 |
| METHARC41 | Methanobacterium alcaliphilum strain NBRC 105226 16S ribosomal RNA gene, partial sequence | 99 |
| METHARC42 | Methanobacterium alcaliphilum strain NBRC 105226 16S ribosomal RNA gene, partial sequence | 99 |
| METHARC43 | Methanomassiliicoccus luminyensis strain B10 16S ribosomal RNA gene, partial sequence | 89 |
| METHARC44 | Methanomassiliicoccus luminyensis strain B10 16S ribosomal RNA gene, partial sequence | 99 |

Table B: Archaeal clone library sequence matches
